# Digital Quantification of Human Eye Color Highlights Genetic Association of Three New Loci

**DOI:** 10.1371/journal.pgen.1000934

**Published:** 2010-05-06

**Authors:** Fan Liu, Andreas Wollstein, Pirro G. Hysi, Georgina A. Ankra-Badu, Timothy D. Spector, Daniel Park, Gu Zhu, Mats Larsson, David L. Duffy, Grant W. Montgomery, David A. Mackey, Susan Walsh, Oscar Lao, Albert Hofman, Fernando Rivadeneira, Johannes R. Vingerling, André G. Uitterlinden, Nicholas G. Martin, Christopher J. Hammond, Manfred Kayser

**Affiliations:** 1 Department of Forensic Molecular Biology, Erasmus University Medical Center, Rotterdam, The Netherlands; 2 Cologne Center for Genomics (CCG), University of Cologne, Cologne, Germany; 3 Department of Twin Research and Genetic Epidemiology, King's College London, London, United Kingdom; 4 Queensland Institute of Medical Research, Brisbane, Australia; 5 Centre for Ophthalmology and Visual Science, Lions Eye Institute, University of Western Australia, Perth, Australia; 6 Department of Epidemiology, Erasmus University Medical Center, Rotterdam, The Netherlands; 7 Department of Internal Medicine, Erasmus University Medical Center, Rotterdam, The Netherlands; 8 Department of Ophthalmology, Erasmus University Medical Center, Rotterdam, The Netherlands; University of Oxford, United Kingdom

## Abstract

Previous studies have successfully identified genetic variants in several genes associated with human iris (eye) color; however, they all used simplified categorical trait information. Here, we quantified continuous eye color variation into hue and saturation values using high-resolution digital full-eye photographs and conducted a genome-wide association study on 5,951 Dutch Europeans from the Rotterdam Study. Three new regions, 1q42.3, 17q25.3, and 21q22.13, were highlighted meeting the criterion for genome-wide statistically significant association. The latter two loci were replicated in 2,261 individuals from the UK and in 1,282 from Australia. The *LYST* gene at 1q42.3 and the *DSCR9* gene at 21q22.13 serve as promising functional candidates. A model for predicting quantitative eye colors explained over 50% of trait variance in the Rotterdam Study. Over all our data exemplify that fine phenotyping is a useful strategy for finding genes involved in human complex traits.

## Author Summary

We measured human eye color to hue and saturation values from high-resolution, digital, full-eye photographs of several thousand Dutch Europeans. This quantitative approach, which is extremely cost-effective, portable, and time efficient, revealed that human eye color varies along more dimensions than the one represented by the blue-green-brown categories studied previously. Our work represents the first genome-wide study of quantitative human eye color. We clearly identified 3 new loci, *LYST*, 17q25.3, *TTC3*/*DSCR9*, in contributing to the natural and subtle eye color variation along multiple dimensions, providing new leads towards a more detailed understanding of the genetic basis of human eye color. Our quantitative prediction model explained over 50% of eye color variance, representing the highest accuracy achieved so far in genomic prediction of human complex and quantitative traits, with relevance for future forensic applications.

## Introduction

The iris functions as the diaphragm of the eye controlling the amount of light reaching the retina. The type, distribution, and amount of pigments in the iris determine eye color [1], [2]. Eye color shows a high degree of variation in people of European ancestry and correlates with latitude within the European continent, which may be explained by a combination of natural and sexual selection [3]. The inheritance of eye color is not strictly Mendelian although blue iris color follows largely a recessive pattern [1]. Genome-wide association studies in people of Europeans decent [4]–[7] have confirmed eye color as a polygenic trait, with the *HERC2*/*OCA2* genes explaining the most of the blue and brown eye color inheritance, whereas other genes such as *SLC2A4*, *TYR*, *TYRP1*, *SLC45A2*, and *IRF4* contribute additionally to eye color variation, albeit with minor effects [8]. These findings increased our understanding of the genetic basis of human pigmentation, and drew attention to their potential applications, such as in forensic sciences [9], [10].

However, all previous genetic studies on human eye color were based on categorical trait information, most often a three-point scale of blue, green-hazel or intermediate, and brown eye color [4]–[6], [11], [12], whereas it is known that in reality iris colour exists in a more continuous grading from the lightest shades of blue to the darkest of brown or black [13]. The use of categorized information from continuous traits is expected to oversimplify the quantitative nature of the trait. Therefore, additional genes contributing to human iris coloration may be identifiable if the full quantitative spectrum of eye coloration could be exploited. To this aim, we digitally quantified continuous eye colors into hue and saturation values from high-resolution, full-eye photographs, and conducted a genome-wide association study in 5,951 Dutch Europeans from the Rotterdam Study genotyped with 550–610,000 single nucleotide polymorphisms (SNPs). Genetic variants with genome-wide significant eye color association were further tested in replication samples of 2,261 participants of the UK Twin Study (TwinsUK) and 1,282 participants of the Brisbane Twin Nevus Study (BTNS) Australia. Finally, we evaluated the predictive value of an updated list of informative SNPs, including interacting ones, on quantitative eye color that is of relevance in forensic applications.

## Results

### Quantitative eye color phenotyping

**Figure 1:**
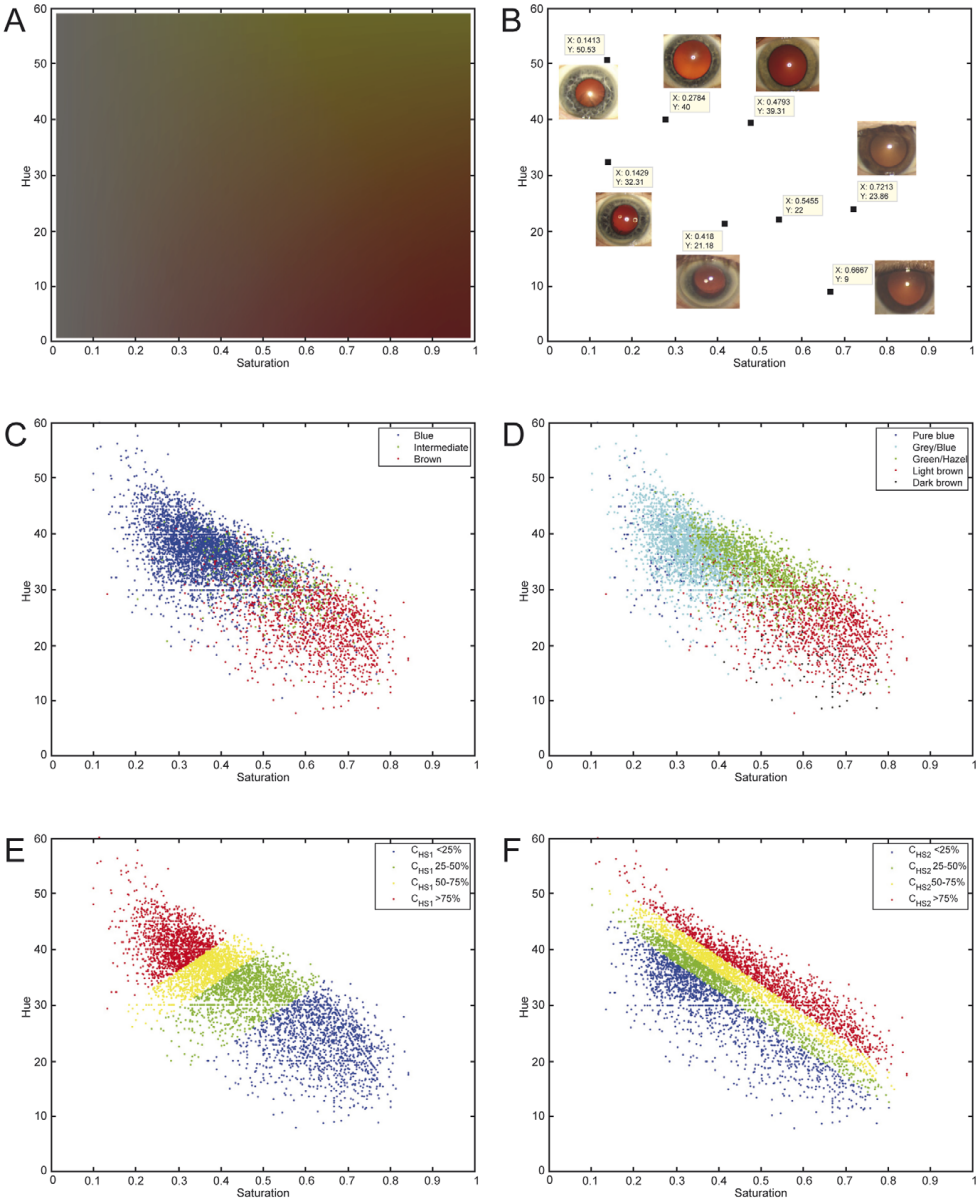
The Hue-Saturation (H-S) eye color space in the Rotterdam Study (RS123). (A) The Hue-Saturation (H-S) eye color space; (B) Example eye photos at their respective position in the H-S space; (C) 3 color categories defined by an ophthalmologist during eye examination and highlighted in the H-S space; (D) 5 categories defined by two researchers from digital full eye size photographs used for digital quantitative extraction of eye colors; (E) 4 quartiles of the 1st principle component C_HS1_; and (F) 4 quartiles of the 2nd principle component C_HS2_; all depicted on the H-S color space.

The discovery sample set included participants of three Rotterdam Study (RS) cohorts (RS1, RS2, and RS3) with a total of 5,951 Dutch European individuals after quality control of genetic and phenotypic data (Table 1). Digitally extracted iris (eye) color was quantified into two interval dimensions hue (H) and saturation (S) (Figure 1A and 1B). H measures the variation in color spectrum, whereas S measures the variation in color purity or intensity. Thus, H and S may serve as representations of the type and the amount of iris pigments. We noticed a high correlation between H and S (r = −0.77), which may have a biological explanation. Eyes classified in three different color categories “blue”, “brown” and “intermediate” by an ophthalmologist during eye examination largely clustered around distinct areas on the HS color space but with considerable overlap (Figure 1C). This is also true for the five color categories graded by reviewing the digital photographs used for eye color quantification in this report (Figure 1D). The overlap between clusters may be expected given the quantitative nature of iris coloration and the variation in color conception. Principal component analysis on z-transformed H and S values revealed two components C_HS1_ and C_HS2_ that accounted for 88.75% and 11.25% of the total quantitative eye color variance (Figure 1E and 1F). Among the 4 quantitative measurements, the C_HS1_ variable showed the highest correlation with the 3-ordinal category variable blue-intermediate-brown.

**Table 1:** Eye color details and demographics of the study subjects.

	RS1		RS2		RS3		TwinsUK		BTNS	
N individuals	2429		1535		1987		2261		1282	
N SNPs (K)	550		550		610					
Age	74.02	8.23	67.65	7.37	56.24	5.80	52.22	12.52	17.19	4.56
Female (%)	59.40		54.40		56.10		89.31		51.48	
Hue	34.86	7.62	32.19	7.25	32.27	6.93	19.22	18.44	27.99	6.38
Saturation	0.40	0.13	0.44	0.14	0.48	0.15	0.47	0.19	0.54	0.24
Blue	68.01		69.51		71.26				53.51	
Intermediate	9.76		4.82		7.50				28.39	
Brown	22.23		25.67		21.24				18.10	
Dark blue	4.12		3.52		2.62					
Grey/light blue	57.93		52.83		41.62					
Green/brown spots	18.98		18.89		31.71					
Light brown	18.03		23.13		22.35					
Dark brown	0.95		1.63		1.71					

Raw values, percentages, or means and standard deviations.

High resolution digital full eye size photos were available in Rotterdam Studies (RS1-3) and Brisbane Twin Nevus Study (BTNS), whereas in the Twin Study from the UK (TwinsUK) only digital full size portrait photos were available with low iris resolution.

### Genome-wide association studies (GWAS)

**Figure 2:**
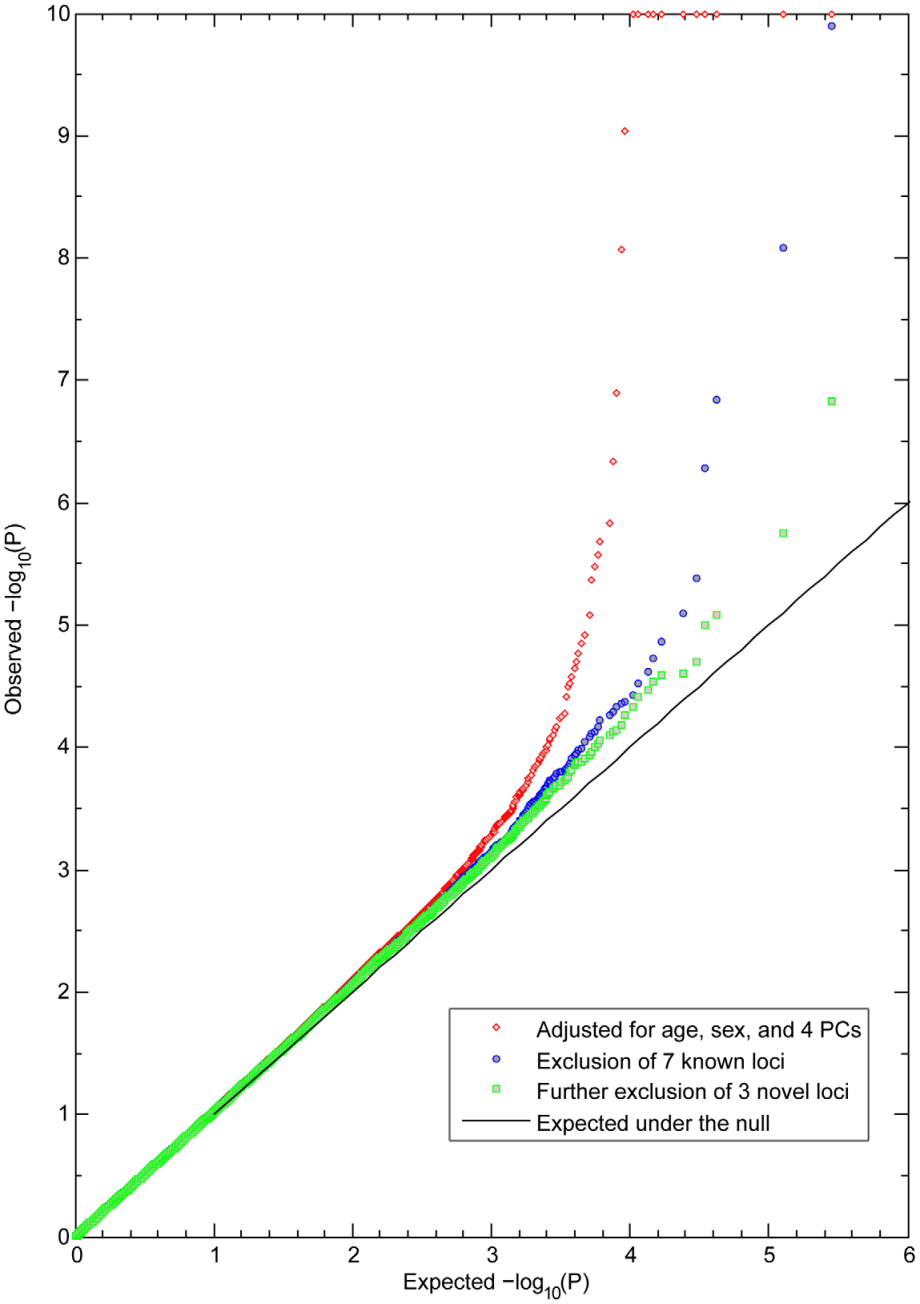
Observed and expected P values for eye color in the Rotterdam Study (RS123). Observed −log_10_ P values in a GWA of C_HS1_ are ranked on the y-axis and plotted against the expected distribution under the null on the x-axis. All P values smaller than 10^−10^ were truncated at 10 at the log scale. The red dots are the P values excluding the effects of sex, age, and population stratification. Blue dots are the P values excluding the effects of 7 genes previously known to be involved in eye color. Green dots are the P values after additionally excluding the effects of 3 newly identified loci, with no more SNPs showing significant association at the genome-wide level.

**Figure 3:**
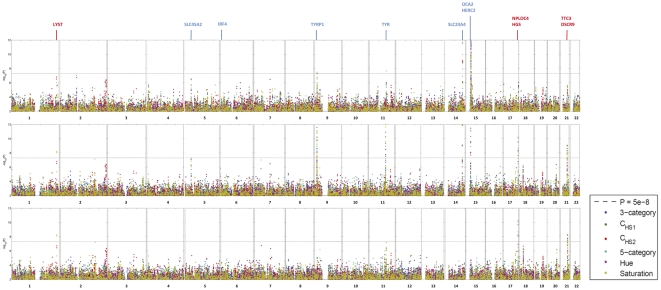
GWA Manhattan plot for quantitative and categorical eye color in the Rotterdam Study (RS123). The −log10 P values for association with 6 eye color traits (hue, saturation, C_HS1_, C_HS2_, 3-category classification, 5-category classification) are plotted for each genotyped SNP according to its chromosomal position (NCBI build 36). The distance between each tick on the x-axis represents 50 Mb. The P values smaller than 10^−12^ are truncated at the level of 12 at the log scale and those greater than 0.01 are not shown. (A) P values are adjusted for age, sex and population stratification, (B) additionally adjusted for the effect of rs12913832 in *HERC2*, the most significantly associated eye color SNP known before, and (C) additionally adjusted for the effect of all 7 previously known eye color associated genes. Previously known eye color genes with genome-wide significant eye color association in the present study are noted using blue text above the figure and genes in the three newly identified loci in the 3rd scan are noted in red.

GWAS in three independent RS cohorts, as well as in the merged dataset (RS123), were carried out for 6 eye color traits i) H, ii) S, iii) C_HS1_, iv) C_HS2_, v) 3-category color classification (“blue”, “brown” and “intermediate”), and vi) 5-category color classification (“pure blue”, “light blue/grey”, “green/mixed with brown spots”, “light brown”, and “dark brown”). Genetic outliers of non-European ancestry were excluded (Figure S1A). No institutional heterogeneity between the three cohorts or residual population sub-stratification was noticed after merging the genotype data (Figure S1B). Inflation factors for all color traits were in the range from 1.02 to 1.03 after adjusting for population sub-stratification. The initial scan of the merged R123 samples for all color traits revealed a sharp deviation between the observed P values and the expected ones under the null hypothesis (Figure 2), mainly due to a very strong effect of the *HERC2* and *OCA2* genes on chromosome 15q13.1 (Figure 3A and Table S1). SNPs in *HERC2* showed the most significant effect on all color traits (rs12913832 P<10^−300^; except for C_HS2_ with P = 0.60) (Figure 3, Table S1), confirming previous findings on categorical eye color information [5]–[6], [11], [14]. In the subsequent scan adjusted for the effect of *HERC2* rs12913832, five other genes known to be involved in eye color (*OCA2*, *SLC2A4*, *TYR*, *TYRP1*, and *SLC45A2*) [4], [7] revealed genome-wide significant eye color association (P<5×10^−8^), and the effect of *IRF4* [7] was confirmed at a somewhat lower significance level (P = 1.4×10^−6^) (Figure 3B). We did not observe a significant effect of *ASIP* on eye color, which is in agreement with our earlier study on categorized eye color [8], and in line with previous findings suggesting that *ASIP* may be more involved in skin pigmentation [4], [15]. Noteworthy, SNPs in the previously known eye color genes *TYRP1*, *TYR*, and *SLC24A4* showed more significant association with quantitative eye color compared with categorical ones (Figure 3B). In the subsequent GWAS adjusted for the effects of all 7 known genes, the P values derived for C_HS1_, H and S still significantly deviated from the expected ones (Figure 2). The tail of deviation was mainly explained by 10 SNPs at 3 new loci 1q42.3, 17q25.3, and 21q22.13 (Table 2, Figure 3C). The association of the three new loci met the genome-wide significance criterion of P<5×10^−8^. The allelic effects of the 10 SNPs were consistent through the 3 independent RS cohorts and were nominally significant (Table 2). No more SNPs were clearly associated with any eye color trait at the genome-wide significant level in an additional scan adjusted for all previously known genes as well as the 3 new loci.

**Table 2:** New SNPs associated with eye color in Rotterdam Studies, and replication analyses in TwinsUK Study and Brisbane Twin Nevus Study (BTNS).

			RS1 (n = 2,429)		RS2 (n = 1,535)		RS3 (n = 1,987)		RS123 (n = 5,951)		TwinsUK (n = 2,261)		BTNS (n = 1,282)		Combined (n = 9,494)	
SNP	EA	Trait	beta	P	beta	P	beta	P	beta	P	beta	P	beta	P	beta	P
1q42.3 *LYST*																
rs3768056	G	S	0.01	5.0E-05	0.01	3.7E-03	0.01	1.3E-03	0.01	7.8E-09	−0.01	5.4E-01	−0.01	1.9E-01	0.01	5.3E-04
rs9782955	T	S	0.01	3.3E-05	0.01	3.6E-03	0.01	1.2E-03	0.01	5.5E-09	0.00	7.5E-01	−0.01	1.5E-01	0.01	3.9E-04
17q25.3 *NPLOC4-HGS*																
rs7219915	T	C_HS1_	0.11	4.1E-05	0.10	1.8E-03	0.10	2.8E-04	0.11	5.9E-11	0.01	8.7E-01	0.10	4.3E-03	0.10	1.5E-13
rs9894429	T	C_HS1_	0.11	2.8E-05	0.07	2.0E-02	0.11	2.8E-05	0.10	2.0E-10	0.01	9.2E-01	0.12	7.0E-04	0.12	8.9E-14
rs12452184	T	C_HS1_	0.10	1.5E-04	0.08	1.2E-02	0.10	2.1E-04	0.10	7.2E-09	0.07	2.7E-01	0.07	6.1E-02	0.07	9.0E-10
21q22.13 *TTC3-DSCR9*																
rs1003719	A	C_HS1_	−0.10	7.2E-05	−0.11	5.6E-04	−0.06	2.8E-02	−0.09	1.9E-08	−0.12	9.1E-04	−0.08	4.1E-02	−0.10	2.3E-10
rs2252893	C	C_HS1_	−0.12	2.4E-06	−0.08	1.0E-02	−0.06	3.4E-02	−0.10	6.0E-09	−0.11	2.3E-03	−0.08	4.1E-02	−0.10	2.3E-10
rs2835621	A	C_HS1_	−0.12	3.2E-06	−0.09	8.0E-03	−0.06	3.1E-02	−0.10	5.0E-09	−0.11	2.3E-03	−0.08	4.1E-02	−0.10	2.3E-10
rs2835630	G	C_HS1_	−0.13	1.2E-06	−0.09	9.2E-03	−0.04	1.2E-01	−0.09	3.1E-08	−0.10	4.8E-03	−0.09	1.8E-02	−0.10	3.6E-10
rs7277820	G	C_HS1_	−0.12	1.5E-06	−0.09	4.9E-03	−0.04	1.0E-01	−0.09	1.5E-08	−0.10	6.9E-03	−0.09	1.5E-02	−0.09	1.4E-08

EA, the effect allele based on which beta was derived.

RS123, merged data of Rotterdam Study 1, 2, and 3.

In TwinsUK SNPs at 17q25.3 were associated with S and C_HS2_ (P<0.02).

**Figure 4:**
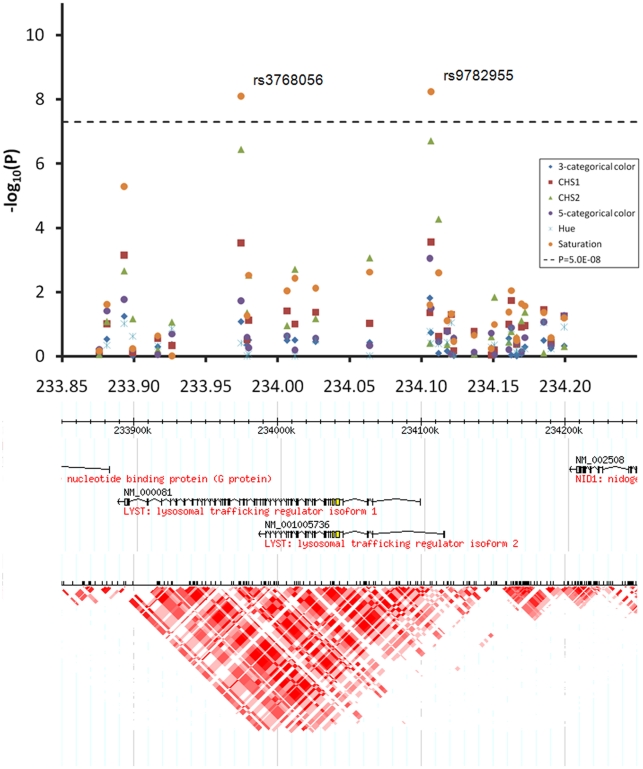
Chromosome 1q42.3 associated with quantitative eye color in the Rotterdam Study (RS123). Regional association plots for 300 Kb surrounding the three newly identified eye color locus on chromosomal 1q42.3. Statistical significance of associated SNPs at each locus are shown on the −log(P) scale as a function of chromosomal position. P values were derived for 6 eye color traits (see figure legend). Genes in the region and LD patterns according to HapMap version 21a CEU samples are aligned bellow. Chromosome 1 233.85–234.25 Mb region includes the *LYST* gene, where SNPs rs3768056 and rs9782955 showed genome-wide significant association with saturation only.

**Figure 5:**
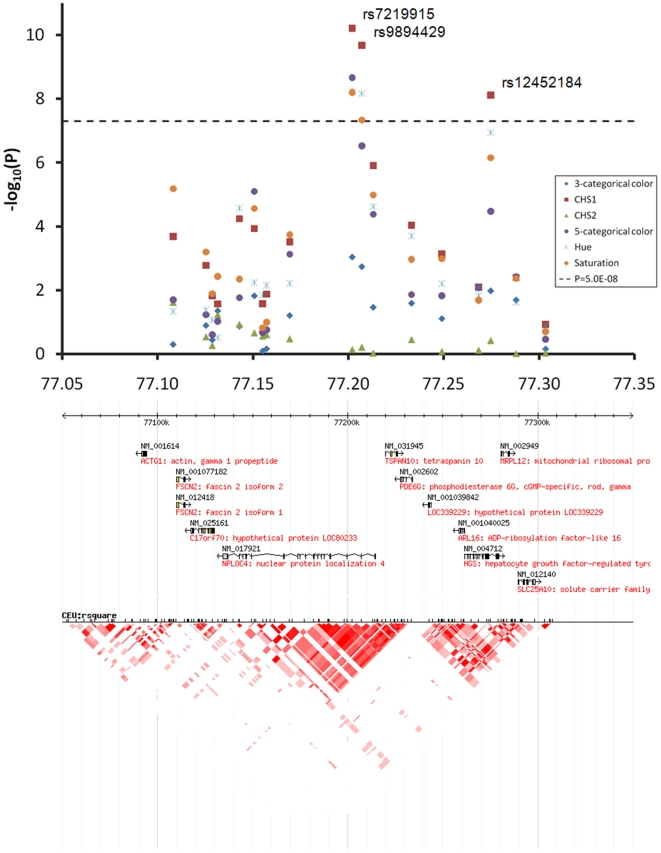
Chromosome 17q25.3 associated with quantitative eye color in the Rotterdam Study (RS123). The chromosome 17 77.05–77.35 Mb region includes multiple small genes, SNPs rs7219915, rs9894429, and rs12452184 showed genome-wide significant association with multiple traits.

**Figure 6:**
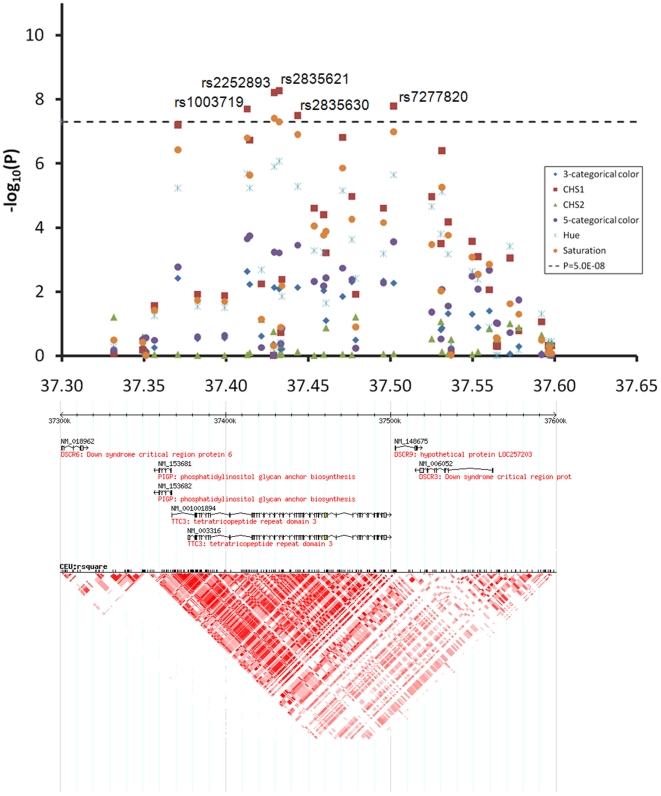
Chromosome 21q22.13 locus associated with quantitative eye color in the Rotterdam Study (RS123). The chromosome 21 37.30–37.65 Mb region includes *DSCR6*, *PIGP*, *TTC3*, *DSCR9*, and *DSCR3* genes, SNPs rs1003719, rs2252893, rs2835621, rs2835630, and rs7277820 showed genome-wide significant association with C_HS1_.

At the 1q42.3 locus two SNPs, rs3768056 and rs9782955, were associated with S at the genome-wide significance level (5.5×10^−9^<P<7.8×10^−9^) (Table 2, Figure 4). Both SNPs are located in introns of the lysosomal trafficking regulator (*LYST*) gene. Note that SNPs at this locus were associated with S but not with H or categorical colors, which is a different phenomenon compared to the other two new loci identified. Three SNPs at 17q25.3 were associated with multiple color traits at the genome-wide significance level and the association with C_HS1_ was the most significant (5.9×10^−11^<P<7.2×10^−9^) (Table 2, Figure 5). The SNP rs7219915 is intronic and rs9894429 exonic of the nuclear protein localization 4 homolog (*NPLOC4*) gene and rs12452184 is intronic of the hepatocyte growth factor-regulated tyrosine kinase substrate (*HGS*) gene. There are multiple small genes in the 17q25.3 region (Figure 5). Five SNPs at 21q22.13 were significantly associated with C_HS1_ (5.0×10^−9^<P<3.1×10^−8^) (Table 2, Figure 6). Four SNPs, rs1003719, rs2252893, rs2835621, and rs2835630, are intronic of the tetratricopeptide repeat domain 3 (*TTC3*) gene, and one, rs7277820, is in the flanking 5′ UTR region of the Down Syndrome Critical Region 9 (*DSCR9*) gene. The *TTC3* and *DSCR9* genes are in the same LD block (Figure 6).

On chromosome 2q37 SNPs rs2070959, rs1105879, rs892839, rs10209564 were associated with C_HS2_ at borderline genome-wide significance (10^−7^<P<10^−6^, Figure 3C). The first 2 SNPs are in the coding region of the UDP glycosyltransferase 1 family (*UGT1A*) gene.

### Replication analyses in TwinsUK and BTNS

Eye color data from the TwinsUK cohort were extracted from digital portrait photographs with limited iris resolution. As these photographs were taken under some variation in daylight and exposure conditions, the trait variance was larger compared with those of RS (H = 19.22±18.44; S = 0.47±0.19; Table 1). This, in combination with smaller sample size, resulted in less significant eye color association detected for the previously known eye color SNPs, such as *HERC2* rs12913832 (RS123: P<1×10^−300^, TwinsUK: P = 1.4×10^−88^), *SLC24A4* rs12896399 (RS123: P = 2.0×10^−23^, TwinsUK: P = 2.1×10^−3^), *TYR* rs1393350 (RS123: P = 1.0×10^−9^, TwinsUK: P = 3.9×10^−2^), and *TYRP1* rs1325127 (RS123: P = 4.0×10^−11^, TwinsUK P>0.05). Despite the considerable loss of statistical power, two of the three regions newly identified here were replicated with significant eye color association in the TwinsUK data. The SNPs at chromosome 21q22.13 locus were replicated with consistent allelic effects (P for C_HS1_ and H<0.01, Table 2). The SNPs at 17q25.3 were associated with S and C_HS2_ (P<0.02, not shown), but not significant with C_HS1_ (0.92<P<0.27, Table 2), which was the most significant association in the RS cohort (P = 5.9×10^−11^). The chromosome 1q42.3 region was not significantly associated with any eye color trait in the TwinsUK data.

Participants of the BTNS cohort were on average much younger (17.19±4.56 years) than the other 2 cohorts (over 50 years) and had more intermediate colored eyes compared with RS (Table 1). The eye photographs from BTNS had similar resolutions and sizes as the ones from RS; however, in contrast to RS they were also taken under some variation in daylight and exposure conditions and the effective sample size was the smallest among the 3 studies. P-values derived from BTNS for the association between eye color and previously known eye color SNPs were somewhat in between those derived from RS and TwinsUK (e.g. P for rs12913832 = 1.26×10^−200^). The newly identified SNPs at 17q25.3 (P for C_HS1_<0.05) and 21q22.13 (P for C_HS1_ and S<0.05) showed significant association with eye color and the betas were consistent with those derived from RS (Table 2). The chromosome 1q42.3 region was not significantly associated with any eye color trait in the BTNS data.

In a combined analysis of all 9494 participants of the RS, TwinsUK, and BTNS cohorts, the association signals at 17q25.3 and 21q22.13 were genome-wide significant (P = 8.9×10^−14^ and P = 2.3×10^−10^, respectively, Table 2), whereas the signal at 1q42.3 did not reach the genome-wide significance threshold (P = 3.9×10^−4^, Table 2), as may be expected from the results of the individual cohorts. Of note, the photos from the 3 study cohorts, from which eye color was digitally extracted, were ascertained based on different approaches (see Methods), and as result, the H and S values showed different means and variance between the 3 cohorts (Table 1). Hence, using all these data in a combined analysis may result in a conservative association signal.

### Eye color prediction

We identified 17 predictors that significantly explained the trait variance, including age and sex, 11 SNPs from 9 genes, and 4 SNP pairs that showed significant interaction effects. For details of interaction analysis, see Text S1 and Figures S2, S3, S4. The 17 predictors together explained 48.87% of the H variance and 56.30% of the S variance in the Rotterdam Study (Table 3). Most predictors had significant effects on both H and S. Exceptions were rs3768056 in *LYST* and the interaction between *HERC2* rs12913832 and *SLC24A4* rs12896399, which were only significant for S, as well as *IRF4* rs12203592, *OCA2* rs728405, and the interaction between *HERC2* rs12913832 and *OCA2* rs728405, which were only significant for H. The main effect of *SLC45A2* rs16891982 is no longer significant when its interaction with rs1800407 was taken into account. The *HERC2* SNP rs12913832 showed, as expected, the strongest predictive power, which alone explained 44.50% of the H and 48.31% of the S variance. Surprisingly, age was identified to be the 2^nd^ strongest predictor of quantitative eye color; the increased age was associated with increased H (ΔR^2^ = 1.17%, P = 8.2×10^−29^) and decreased S (ΔR^2^ = 5.03%, P = 1.4×10^−131^). The 3 newly identified loci together explained 0.53% and 0.73% and the identified SNP-SNP interactions explained 0.75% and 0.72% of the H and S variance, respectively. Gender showed a small effect on H (ΔR^2^ = 0.04%) and S (ΔR^2^ = 0.09%), although statistically significant (P<0.04). After adjusting for the effects of the 17 predictors, the summary variance explained by the remaining SNPs was negligible (ΔR^2^<0.01%). These 17 identified predictors explained 56.2% of S and 11.1% of H variance in BTNS as well as 28.5% of S and 4.1% of H in TwinsUK.

**Table 3:** Predicting quantitative eye color in Rotterdam Studies.

		Hue						Saturation					
Predictors	Gene	Beta	se	ΔF	P value	ΔR^2^ %	rank	Beta	se	ΔF	P value	ΔR^2^ %	rank
Constant		34.31	1.32					0.550	0.011				
Female		0.29	0.14	4.2	4.2E-02	0.04	16	0.009	0.003	11.8	6.0E-04	0.09	14
Age per 10 yr		0.71	0.07	125.4	8.2E-29	1.17	2	−0.030	0.001	627.5	1.4E-131	5.03	2
rs3768056G	*LYST*	---	---	---	NS	---	---	0.013	0.002	37.9	8.0E-10	0.29	6
rs16891982C	*SLC45A2*	---	---	---	NS	---	---	---	---	---	NS	---	---
rs12203592A	*IRF4*	−2.10	0.51	17.9	2.4E-05	0.16	10	---	---	---	NS	---	---
rs1325127G	*TYRP1*	−0.54	0.11	23.5	1.3E-06	0.21	7	0.012	0.002	40.9	1.7E-10	0.32	5
rs1393350A	*TYR*	0.38	0.11	13.9	1.9E-04	0.12	14	−0.011	0.002	34.0	5.9E-09	0.26	7
rs12896399C	*SLC24A4*	−0.46	0.10	21.9	3.0E-06	0.20	9	0.059	0.005	125.0	9.9E-29	0.98	3
rs728405C	*OCA2*	−1.38	0.41	11.8	6.0E-04	0.10	15	---	---	---	NS	---	---
rs1800407G	*OCA2*	7.69	1.30	20.7	5.6E-06	0.18	12	−0.062	0.010	19.6	9.8E-06	0.15	11
rs1129038C	*HERC2*	−4.55	0.43	96.2	1.6E-22	0.88	3	0.027	0.008	12.3	4.5E-04	0.09	13
rs12913832A*	*HERC2*	−9.91	0.74	4665.6	<1E-300	44.50	1	0.258	0.012	5438.3	<1E-300	48.31	1
rs9894429A	*NPLOC4*	0.55	0.10	33.1	9.2E-09	0.30	5	−0.009	0.002	25.0	5.9E-07	0.19	9
rs7277820G	*DSCR9*	−0.45	0.10	25.7	4.2E-07	0.23	6	0.010	0.002	33.6	7.1E-09	0.25	8
rs1800407G_ rs16891982C		−5.25	1.09	23.7	1.1E-06	0.21	13	0.025	0.006	15.8	7.3E-05	0.12	12
rs12913832A_rs12203592A		2.11	0.36	33.9	6.1E-09	0.31	4	−0.011	0.002	23.3	1.4E-06	0.18	10
rs12913832A_rs728405C		1.13	0.27	22.5	2.1E-06	0.20	8	---	---	---	NS	---	---
rs12913832A_rs12896399C		---	---	---	NS	---	---	−0.030	0.004	58.9	1.9E-14	0.46	4
Total						48.79						56.72	

NS: not significant.

Beta, se, and P values were derived in RS1 and RS2 cohorts, R^2^ changes were estimated in RS3 cohort.

The interaction terms are defined at the multiplicative scale.

*rs12913832 A allele is modeled to have a dominant effect, allelic effects in other SNPs are modeled additively.

The main effect of rs16891982C is not significant when the interaction term is included.

We also used the 17 predictors for 3 or 5 categorical eye color prediction based on a multinomial logistic regression model. The prediction accuracy was measured by the Area Under the receiver operation Curve (AUC). The accuracy in predicting 3-category eye color was 0.92 for blue, 0.74 for intermediate, and 0.93 for brown, which reflects a slight but statistically significant (P = 2.7×10^−4^) improvement compared to our previous attempt using 15 SNPs from 8 genes (AUC 0.91 for blue, 0.73 for intermediate, and 0.93 for brown) [8]. Excluding the non-genetic predictors age and gender from the model had no major impact on the prediction accuracy of categorical eye color (ΔAUC<0.01 for any color category). Notably, predicting 5 eye colors was category-wise less accurate compared to the 3-category prediction (AUC 0.72 for pure blue, 0.82 for light blue/grey, 0.66 for green/mixed, 0.93 for light brown, 0.89 for dark brown), which may not be unexpected as by increasing the number of categories in the phenotype classification the uncertainty of assignment also increases.

## Discussion

Using digitally-quantified continuous eye color information, extracted from high-resolution full eye size pictures, we were able to improve the power of finding genetic associations as evident from seeing SNPs in some known eye color genes with more significant association with quantitative than categorical eye color. The gain of power also allowed us to identify 3 new loci, which add substantially to the previously available list of seven genes and provide additional insights into the genetic origins of human pigmentation. Fine-resolution phenotyping may therefore serve as an important alternative strategy for finding genes involved in complex traits to simply increasing sample size, which represents the main trend of current GWA studies in humans.

All SNPs associated with eye color at 1q42.3 are located in the *LYST* gene. Mutations in the *LYST* gene are involved in Chediak-Higashi and exfoliation syndromes characterized by iris pigmentation dispersion, transillumination and other defects [16]. Mice studies showed that *LYST* mutations reproduced the iris defects of human exfoliation syndrome [17]. Furthermore, a study of coat colour in cattle showed that *LYST* may influence the intensity of pigment within coat colour categories, e.g., dark grey to light grey, but do not result in color type changes, e.g., grey to red or black [18]. These authors suggested that allelic variation in this gene, possibly not associated with illness, could underlie the different shades of colours observed in the partially diluted colour. Our results in the Rotterdam Study are in perfect agreement with their conclusion. Also, the *LYST* gene was identified in two studies with evidence for positive selection when comparing continental populations that strongly differ in pigmentation phenotypes [19]. This provides additional arguments that the gene is involved in human pigmentation traits [2]. Noteworthy, the SNPs in *LYST* gene were associated at genome-wide significance with saturation only but were not even nominally significant with hue. This finding underlines the relevance of our approach to separately analyze the H and S dimensions, which are likely to involve independent biological bases. The failure to replicate the 1q42.3/*LYST* findings in the TwinsUK and BTNS studies may be explained by a combination of factors related to the smaller sample size, the relatively small effect size (smallest of the 3 loci described in this manuscript), as well as some limitations in the photographs. Lighting conditions and background color were not standardized for the TwinsUK and BTNS cohorts, and picture resolution in the TwinsUK study was much lower than in the other two studies, reducing accuracy of H and S estimation. 1q42.3 was the only region that did not reach genome-wide significance in the combined analysis - again likely to be a result of small effect size, but also that the detected association was with S, whereas both other regions expressed association with C_HS1_, which may be less affected by noise. Although the signals detected at 1q42.3 in the Rotterdam Study may represent a false positive finding, the abundant evidence from animal studies and from human evolutionary studies suggest that *LYST* is likely to influence subtle variation in the amount of pigmentation that requires high precision measurements to be detectable.

The replicated significant association at chromosome 17q25.3 locus, which also showed genome-wide significance in the combined analysis, was detected for SNPs located in the *NPLOC4* and *HGS* genes. There are, however, multiple small genes in this region, including *ACTG1*, *FSCN2*, *C17orf70*, *NPLOC4*, *TSPAN10*, *PDE6G*, *LOC339229*, *ARL16*, *HGS*, *MRPL12*, and *SLC25A10*. At this moment it is difficult to clearly affiliate a functional unit to the association signal observed. Based on current knowledge, *PDE6G* may be the best candidate gene for the association signal observed. Mutations in *PDE6G* cause autosomal recessive retinitis pigmentosa [20], in which the dysfunction in retinal pigment epithelium is typical.

The chromosome 21q22.13 locus, which we identified with replicated significant eye color association, and also in the combined analysis, contains several genes including the Down Syndrome Critical Region 3 (*DSCR3*), 6 (*DSCR6*), 9 (*DSCR9*), tetratricopeptide repeat domain 3 (*TTC3*), and phosphatidylinositol glycan anchor biosynthesis (*PIGP*) genes. The SNPs showing significant association with eye color were in the *TTC3* and *DSCR9* genes. Both genes are in the same high linkage disequilibrium region. It is known that trisomy of the chromosomal 21q22 region leads to Down syndrome in which so called Brushfield spots are often observed [21]. Brushfield spots are small white or grayish/brown spots on the periphery of the human iris due to aggregation of connective tissue, a normal iris element. These spots are normal in children but much more frequently (up to 78%) observed in newborn Down Syndrome patients [22]. Also, they are much more likely to occur in patients of European origin, where eye color variation is observed, compared to patients of Asian ancestry with homogeneous brown eyes [23]. Further, the *DSCR9* gene, encoding functionally unknown proteins, was found a new gene in the primate lineage during evolution and exclusive to primate genomes [24]. We therefore hypothesize that genetic variants in *DSCR9* or nearby genes may influence the aggregation of connective tissue of normal iris resulting in different iris color appearance, and extreme forms of variation, e.g., via trisomy, lead to Down Syndrome. It has been suggested that the development of the iris and brain are linked, speculatively via genetic pathways that may also involve pigment production [25].

There remained several residual signals over the genome at borderline genome-wide significant association with eye color in the Rotterdam Studies. Such signals may represent false positive results or genes with true but small effects requiring a larger sample for detecting unambiguous associations or iris color phenotypes of even more detailed characterization as obtainable here. Most notably is the association identified at 2q37; this region includes the *UGT1A* gene encoding a UDP-glucuronosyltransferase, an enzyme of the glucuronidation pathway that transforms bilirubin into water-soluble metabolites. Variants in this gene influence bilirubin plasma levels [26], and were suggested to cause Gilbert's syndrome [27], which is the most common syndrome known in humans characterized with mild and harmless jaundice characterized by a yellowish discoloration of the skin. Interestingly, SNPs in the *UGT1A* gene were most significantly associated with C_HS2_, a dimension that is uncorrelated with the blue-brown variation represented by C_HS1_, indicating that C_HS2_ may represent the variation in yellowish pigments.

The *HERC2/OCA2* genes showed some “masking” effects over *SLC24A4*, *SLC45A2* and *IRF4* genes (Figure S4) that significantly improved the prediction accuracy. However, it remains uncertain if these interactions are truly genetic or confounded by other factors. For example, high melanin concentration in the frontal iris epithelia may block the color variation in the inner layers from being measurable, which may lead to statistically significant interactions. Still, not all genes showed interaction with *HERC2/OCA2* and some of the interactions are specific for the H or S dimension. These findings are of interest for further functional studies.

Our prediction model explained 49–56% of the trait variance in the Rotterdam Study. To our knowledge these values represent the highest accuracy achieved so far in genomic prediction of human complex and quantitative traits [28]. We used non-overlapping samples in building and evaluating the prediction model, and this may lead to slightly conservative R^2^ estimates compared with the methods based on cross validations. Also note that these R^2^ estimates are not equivalent to the ones from linkage-based studies or logistic models. The fact that the identified 17 predictors explain less trait variance in TwinsUK may be addressed by the quality limitations in the photographs available. In both TwinsUK and BTNS the variance explained for H was much lower than that for S. This is most likely because the light conditions were not standardized when the photographs were taken in these two cohorts available for replication analyses. Given that the newly identified genetic variants together explained less than 2% of the trait summary variance, we do not expect that additional but unknown genetic variants may account for an essential portion of the unexplained variance. The color of the eye as perceived from the outside was the main outcome of this study, whereas the pigmentation genes by definition have a more direct effect on the melanin content. However, so far it is unclear if probing deeper into endophenotypes, e.g., directly measuring melanin content using biochemical methods, is going to reduce the unexplained variance, as we have also shown that there are regions putatively associated to eye colour but not clearly involved in the melanin pathways.

Using the 17 predictors for 3-categorical color prediction slightly improved the accuracy compared to our previous attempt using 15 SNPs from 8 genes. The 5-category model had little power in differentiating “pure blue” from “light blue/grey”, and “dark brown” from “light brown” categories, which are more likely to be consequences of differences in tissue structure than chemical composition [1]. The proposed quantitative prediction model may be helpful as an investigative tool in forensic applications, i.e. to better trace unknown suspects in cases where conventional DNA profiles from crime scene samples do not match those of known suspects including those already in criminal DNA databases [9]. Instead of a verbal statement on categorical eye color, which is prone to subjective imagination and is expected to result in inter-individual differences on the actual eye color in question when used to trace unknown persons, our quantitative prediction approach results in a more precisely defined eye color outcomes. For forensic practice we envision that results from DNA-based quantitative eye color prediction tests will be provided as standardized color charts or as computer-based color prints, which could also include uncertainty intervals expressed in colors, hence providing a small range of the most likely colors a DNA sample donor's irises may have. Therefore quantitative eye color prediction is expected to enhance the success rate of tracing unknown individuals according to eye color in forensic applications compared with categorical eye color prediction suggested previously [10]. Our data also demonstrate that eye color saturation declines substantially in elderly people, further emphasizing the gain in power by using a quantitative approach. Age was significant in each of the 3 RS cohorts as well as in the UK and Australia replication cohorts. Thus, its effect on eye color is unlikely a reflection of sample composition and we speculate its effect may share some biological pathways involved in the graying of hair color. Future studies aiming to identify biomarkers for age prediction may further improve the eye color prediction accuracy.

In this study we focused on quantitative H and S dimensions, which may reflect the variation in type and amount of iris pigmentation, whereas the distribution of pigmentation is less covered by these measures. For example, some irises are characterized by an inner brown ring surrounding the pupil and blue/gray color at the outer part of the iris. Such traits reflecting the variation in pigmentation distribution, if measured quantitatively, may be useful for a further and even more detailed understanding of eye color genetics.

Using the example of eye color we have demonstrated that employing quantitative phenotype information about a complex trait in GWA analysis allows detection of new genetic variants. The three new regions and the new genetic interactions identified here as being involved in human quantitative eye color variation may serve as guides for future studies exploring the functional basis of human pigmentation. Finally, our findings are relevant for predicting eye color in applied areas of science such as in forensics.

## Methods

### Rotterdam Study

The Rotterdam Study (RS) is a population-based prospective study including a main cohort and 2 extensions. The RS1 [29] is ongoing since 1990 and included 7,983 participants living in Rotterdam in The Netherlands. The RS2 [30] is an extension of the cohort, started in 1999 and included 3,011 participants. The RS3 [31] is a further extension of the cohort started in 2006 and included 3,932 participants. The participants were all examined in detail at baseline. Collection and purification of DNA have been described in detail previously [6]. Each eye was examined by slit lamp examination by an ophthalmological medical researcher, and iris color was graded by standard images showing various degrees of iris pigmentation. Three categories of iris color (blue, intermediate, and brown) were distinguished based on predominant color and the amount of yellow or brown pigment present in the iris. Additionally, digital full eye size photographs of the anterior segment were obtained with a Sony HAD 3CCD color video camera with a resolution of 800×600 pixel for each of three colors (Sony Electronics Inc., New York, NY) mounted on a Topcon TRC-50EX fundus camera (Topcon Corporation, Tokyo, Japan) after pharmacologic mydriasis (tropicamide 0.5% and phenylephrine 5%). The procedure of pharmacologic mydriasis (dilation of the pupil) was employed because the initial target for taking these pictures was the retina. The treatment makes the area of visible iris tissue smaller (Figure 1B), and, thus, these images were not initially optimized for iris color examination. However, this procedure had little influence on the precision of the color measurements given the large number of the pixels in iris part. Two independent researchers additionally reviewed these images on a monitor with standard settings and graded the eye color into five categories, “pure blue”, “light blue/grey”, “green/mixed with brown spots”, “light brown”, and “dark brown”. The Medical Ethics Committee of the Erasmus University Medical Center approved the study protocol, and all participants provided written informed consent. The current study included in total of 5,951 RS participants who had both genotypic information and eye photos.

### TwinsUK

The TwinsUK cohort is a volunteer cohort of 10,000 same-sex monozygotic and dizygotic twins recruited from the general population (http://www.twinsUK.ac.uk). They have been extensively phenotyped, and gradeable portrait images (digitized from Polaroid photographs and digital photographs), with GWAS information, were available for 2,261 subjects. The study was reviewed by the St Thomas' Hospital Local Research Ethics Committee, and subjects were included after fully informed consent.

### BTNS Australia

Adolescent twins, their siblings and parents have been recruited over sixteen years into an ongoing study of genetic and environmental factors contributing to the development of pigmented nevi and other risk factors for skin cancer as described in detail elsewhere [32]. The proband twins were recruited at age twelve years via schools around Brisbane, Australia, and followed up at age fourteen. Iris colour was scored by a trained nurse. Iris photographs were taken for all twins using a 13.6 megapixel digital camera (Sony Cybershot W300) using a flash. The camera was placed 5–7 cm in front of the eye to be photographed. Images were cropped in-camera to show only the iris, and the cropped 5 megapixel image stored for later processing. BTNS photos were similar with those from RS in term of sizes and resolutions. The pupils were not dilated so more iris area was available to score per individual. However, these photos were taken under some variation in day light conditions and exposure levels. Principal components analysis of Illumina 610k GWAS data for all participants allowed identification of ancestry outliers and these were removed before further analysis so that the sample here is of exclusively northern European origin. All participants gave informed consent to participation in this study, and the study protocol was approved by appropriate institutional review boards. The current study includes 1,282 participants with eye photographs and GWAS information.

### Eye color quantification

To measure colors quantitatively, we first compared several models in representing iris color including the RGB, CIE Lab, CIE XYZ and HSB/HSV models. We chose the HSB model where H stands for hue, S for saturation, and B for brightness. Under a fixed B, HS can be viewed as a color pie where H represents the variation of the color type, ranging from 0°–360° for all human detectable true colors, and the radius S represents the purity or intensity of the color, ranging from 0 to 1. The brightness or luminance is measured by B, a separate dimension that was removed from genetic analysis since it is sensitive to the lighting conditions when a photo is taken. The HSB color model suits well the current application because (1) the perceptual difference in it is uniform, (2) H and S values are invariant to brightness, (3) H and S may represent the type and the amount of iris pigments and (4) H and S values can be directly translated to true colors.

We developed a simple algorithm to automatically retrieve iris colors from the RS eye photos. Starting at the center of an image where the pupil is located, the algorithm samples pixels along multiple radii that cross the pupil, the iris, and the white of the eye in that sequence. The color intensity distribution of the sampled pixels follows a characteristic shape, based on which, the algorithm determines the starting and ending points of the iris by means of edge detection. It then connects all detected edge points by fitting an inner and an outer ellipse. The region between the inner and outer ellipse is considered as the iris region. Median RGB values of the pixels in the iris region were retrieved from each image and transformed to HS values according to standard formulas. The image processing procedures were programmed using Matlab 7.6.0 (The MathWorks, Inc., Natick, MA).

We noticed minor discordances between digital quantification and expert classification; 0.25% (58) “brown” eyes appeared in the blue area of the HS space (H>35 and S<0.45) whereas 1.65% (98) “blue” eyes were in the brown area (H<30 and S>0.55) (Figure 1). Most of these are due to expert misclassification. We kept the color categories of these individuals in the prediction analysis for a fair comparison with our previous prediction results that also allow a certain degree of sampling uncertainty.

Due to significant differences between RS eye photographs and TwinsUK portrait photographs, we preprocessed TwinsUK photographs by correcting R, G, B channels of each photo using 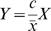, where 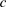 is the channel mean of all photos, 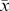 is the channel mean of each photo and 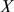 is the matrix of the raw channel values of all pixels in that photo. We then applied the iris color retrieval algorithm on the TwinsUK photographs where the pupil was centralized manually.

We applied the iris color retrieval algorithm on the BTNS full-size eye photographs. BTNS photos were similar with those from RS in term of sizes and resolutions but were also taken under various day light conditions. The resultant distribution on the Hue dimension was not normal with a cluster of samples having low values. The mean correction technique used in TwinsUK data could not be applied because the iris part composed a significant portion of the image. We therefore excluded 66 samples with H<20 from the BTNS data.

### Genotyping and quality control

In RS1 and RS2, genotyping was carried out using the Infinium II HumanHap550K Genotyping BeadChip version 3. Complete information on genotyping protocols and quality control measures for RS1 and RS2 have been described previously [33], [34]. In RS3, the genotyping method followed tightly those of RS1 and RS2 but using a denser array, the Human 610 Quad Arrays of Illumina. We excluded individuals with a call rate <97.5%, gender mismatch with typed X-linked markers, excess autosomal heterozygosity >0.33, duplicates or 1st degree relatives identified using IBS probabilities, and outliers using multi-dimensional scaling analysis with reference to the 210 HapMap samples (Figure S1A). Further excluding individuals without eye photos from all cohorts left 2429 individuals in RS1, 1535 in RS2, and 1987 in RS3 (Table 1). Genome-wide imputation in RS3 also followed tightly the methods used in RS1 and RS2 as described in detail previously [34]. Genotypes were imputed using MACH [35] based upon phased autosomal chromosomes of the HapMap CEU Phase II panel (release 22, build 36), orientated on the positive strand. The scripts developed for this project are freely available online. In total of 2543887 SNPs passed quality control. DNA samples from the TwinsUK registry genotyped using the Hap317K chip (Illumina, San Diego, California, USA). Quality control at individual and SNP levels were described in detail previously [36]. DNA samples from the BTNS were genotyped by the Scientific Services Division at deCODE Genetics, Iceland (http://www.decode.com/genotyping/) using the Illumina 610-Quad BeadChip. Additional genotyping for SNPs within known pigmentation genes was conducted using Sequenom as described in detail previously [37].

### GWA analysis

GWA analysis was conducted in RS1, RS2, and RS3 separately as well as in the merged data set RS123. The genotypes were merged according to the annotation files provided by Illumina on the positive strand. Pair-wise identity by state (IBS) matrix between individuals in RS123 was recalculated by using a subset of pruned markers (50,000 SNPs) that are in approximate linkage equilibrium. Principle components were re-derived using multidimensional scaling analysis of the 1-IBS matrix. The potential institutional heterogeneity between the three RS data sets and residual population stratification were checked by plotting the first 2 principal components (Figure S1B). The effects of sex, age, and 4 main principal components on eye color traits were regressed out prior to GWA analysis. Association was based on a score test of the additive effect of the minor allele and the χ^2^ value with 1df was derived. Inflation factors were derived for each trait and were used to adjust the χ^2^ values. The distribution of observed P values was inspected using Q-Q plots against the P values from the null χ^2^ distribution with 1df. P values smaller than 5×10^−8^ were considered to be genome-wide significant. A subsequent scan is performed on the residuals excluding the effects of the significant SNPs in a previous scan, until no more significant SNP is identified. All significant SNPs were further examined using linear regression for quantitative traits and multinomial logistic regression for categorical traits, where sex, age, and the 4 principal components were adjusted as covariates. GWA analyses were conducted using R library GenABEL v1.4-3 [38] for genotyped SNPs and PLINK v1.07 [39] for imputed data. Haplotype and LD analysis were conducted for the regions of interest using Haploview v4.1 [40]. Replication analysis in TwinsUK and BTNS were conducted using the score test implemented in Merlin [41], which took account of relatedness.

### Prediction analysis

We performed a multivariate analysis and present a linear model for predicting quantitative human eye color. A total of 70 predictors were analyzed, including the 64 SNPs (Table S1), the 4 SNP-SNP interaction terms identified in the interaction analysis (see Text S1 for details), age, and sex. The predictors included in the final model were selected by iteratively including the next ranked predictor that reduces the Akaike information criterion [42] value of the model. The predictors and model parameters were derived in the RS1 and RS2 cohorts and subsequently used to predict eye color H and S in the RS3 cohort. The prediction accuracy was evaluated using R^2^, the variance of H and S that were explained by the predictors in RS3. The genotype of rs12913832 was binary coded as 0 representing the GG genotype and 1 representing the GA or AA genotypes, whereas the genotypes of other SNPs were coded as 0, 1 and 2 number of the minor alleles.

Multinomial logistic regression was used for categorical prediction as described previously [8]. Categorical prediction was evaluated using AUC. Interaction analysis, prediction modeling and evaluation procedures were scripted in Matlab v7.6.0 (The MathWorks, Inc., Natick, MA).

## Supporting Information

Figure S1 Genotype quality control. (A) Genotypes from 120 HapMap Phase 2 subjects were merged with the RS3 samples. QCs of RS1 and RS2 samples have been described in detail previously. The first 2 principal components derived from multidimensional decomposition analysis of the 1-IBS matrix are depicted. Blue circles represent the HapMap European (CEU) samples, green circles are the HapMap East Asian (CHB+JPT) samples, and red circles represent the HapMap West African (YRI) samples. Black and Grey circles are samples from RS3. In total 112 RS3 samples outside of 4 standard deviations of the principle component of the CEU samples were removed. (B) RS1, RS2, and RS3 samples were merged after excluding outliers in separate quality control procedures. The first 2 principal components are depicted. Red circles are the RS1 samples, blue circles are RS2 samples, and green circles are the RS3 samples. No outliers were identified.
(1.10 MB TIF)

Figure S2 SNP interaction analysis. Pair-wise SNP-SNP interactions of 64 SNPs preselected from known eye color genes and in 3 novel loci identified in the current study. SNPs are indexed according to Table S1, sorted according to chromosome and physical positions. The high LD regions include *LYST* (SNPs 1–2), *SLC45A2* (3–4), *IRF4* (5–6), *TYRP1* (7–16), *TYR* (17–23), *SLC24A4* (24–27), *OCA2/HERC2* (28–57), 17q25.3 (58–59), *TTC3/DSCR9* (60–64). The lower right triangle represents the significance of interaction on the −log^10^(P) scale; all P values smaller than 10^−10^ are truncated at 10^−10^. The upper triangle are the linkage disequilibrium r^2^ values×10. (A) Hue, (B) Saturation.
(2.95 MB TIF)

Figure S3 The effect of LD on SNP interaction analysis. 10,000 pairs of SNPs in LD (r^2^>0.5) and 10,000 pairs of SNPs not in LD (r^2^<0.01) were randomly selected over the genome and tested for interaction with the permutated color traits using F-test specified in the method section. The observed P values on the −log^10^(P) scale derived without LD (blue circle) or with LD (red plus) are plotted against the expected ones under the null distribution of no interaction.
(0.39 MB TIF)

Figure S4 Significant SNP interactions on eye color. SNPs having significant interaction effect on eye color are depicted using box-and-whisker diagrams. Color H and S distributions are grouped by cross genotypes of 2 interacting SNPs. Distribution summaries include min-max range (black dotted vertical line), lower-upper 25% quartile range (blue box), and median (red line). Observations outside of 1.5 folds of the quartile range are indicated using red pluses.
(0.34 MB TIF)

Table S1 SNPs ascertained for pair-wise interaction analysis and P values from single SNP analysis in the Rotterdam Study (RS123).
(0.11 MB DOC)

Text S1 Interaction analysis.
(0.04 MB DOC)
