# Supplementary figures and images for: Digital Quantification of Human Eye Color Highlights Genetic Association of Three New Loci
Source: PLoS Genet. 2010 May 6;6(5):e1000934. doi: 10.1371/journal.pgen.1000934 (PMC2865509; doi:10.1371/journal.pgen.1000934)

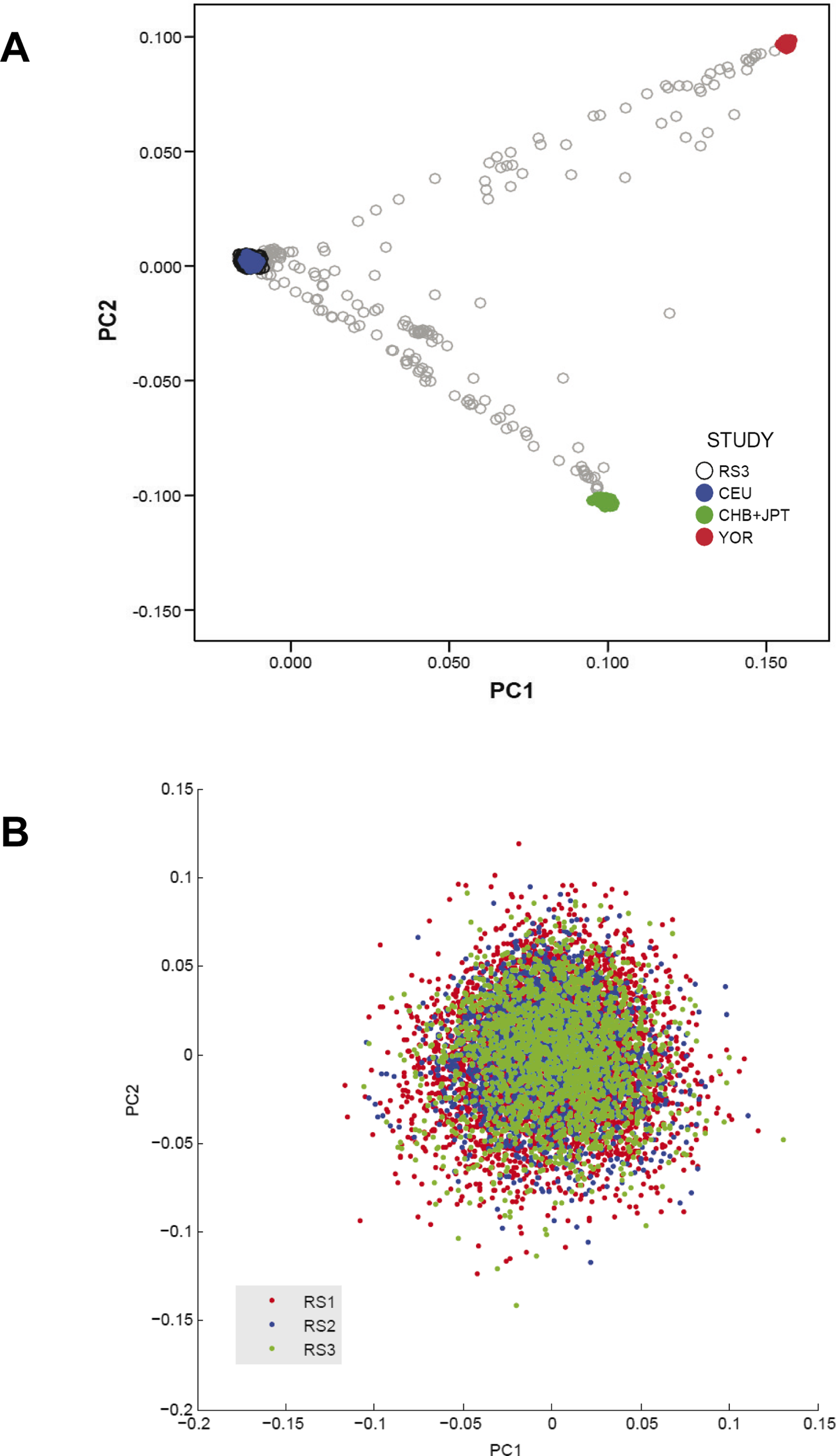

Supplement: Figure S1 — Genotype quality control. (A) Genotypes from 120 HapMap Phase 2 subjects were merged with the RS3 samples. QCs of RS1 and RS2 samples have been described in detail previously. The first 2 principal components derived from multidimensional decomposition analysis of the 1-IBS matrix are depicted. Blue circles represent the HapMap European (CEU) samples, green circles are the HapMap East Asian (CHB+JPT) samples, and red circles represent the HapMap West African (YRI) samples. Black and Grey circles are samples from RS3. In total 112 RS3 samples outside of 4 standard deviations of the principle component of the CEU samples were removed. (B) RS1, RS2, and RS3 samples were merged after excluding outliers in separate quality control procedures. The first 2 principal components are depicted. Red circles are the RS1 samples, blue circles are RS2 samples, and green circles are the RS3 samples. No outliers were identified. (1.10 MB TIF) [file pgen.1000934.s001.tif]

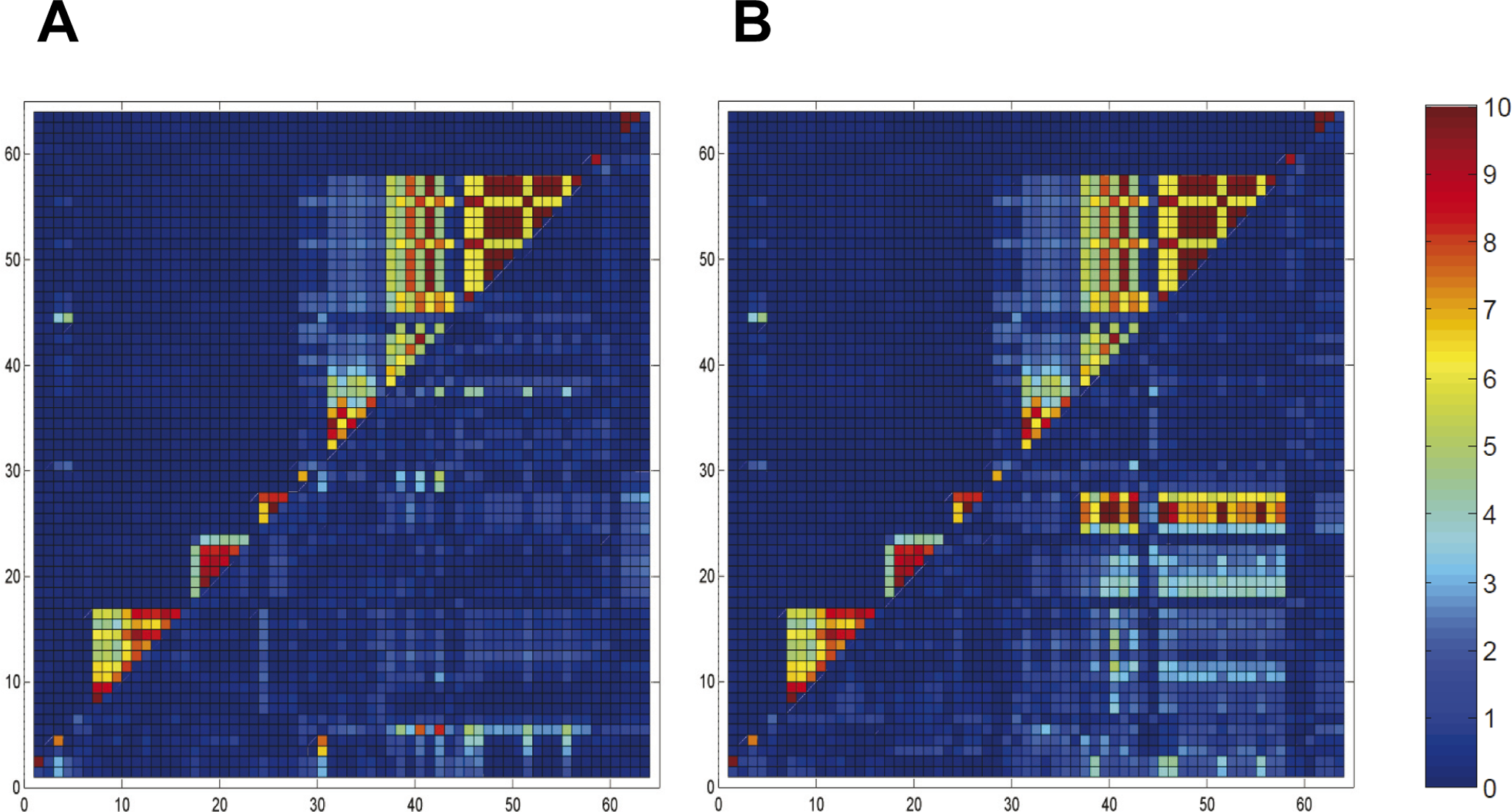

Supplement: Figure S2 — SNP interaction analysis. Pair-wise SNP-SNP interactions of 64 SNPs preselected from known eye color genes and in 3 novel loci identified in the current study. SNPs are indexed according to Table S1, sorted according to chromosome and physical positions. The high LD regions include LYST (SNPs 1–2), SLC45A2 (3–4), IRF4 (5–6), TYRP1 (7–16), TYR (17–23), SLC24A4 (24–27), OCA2/HERC2 (28–57), 17q25.3 (58–59), TTC3/DSCR9 (60–64). The lower right triangle represents the significance of interaction on the −log10(P) scale; all P values smaller than 10−10 are truncated at 10−10. The upper triangle are the linkage disequilibrium r2 values×10. (A) Hue, (B) Saturation. (2.95 MB TIF) [file pgen.1000934.s002.tif]

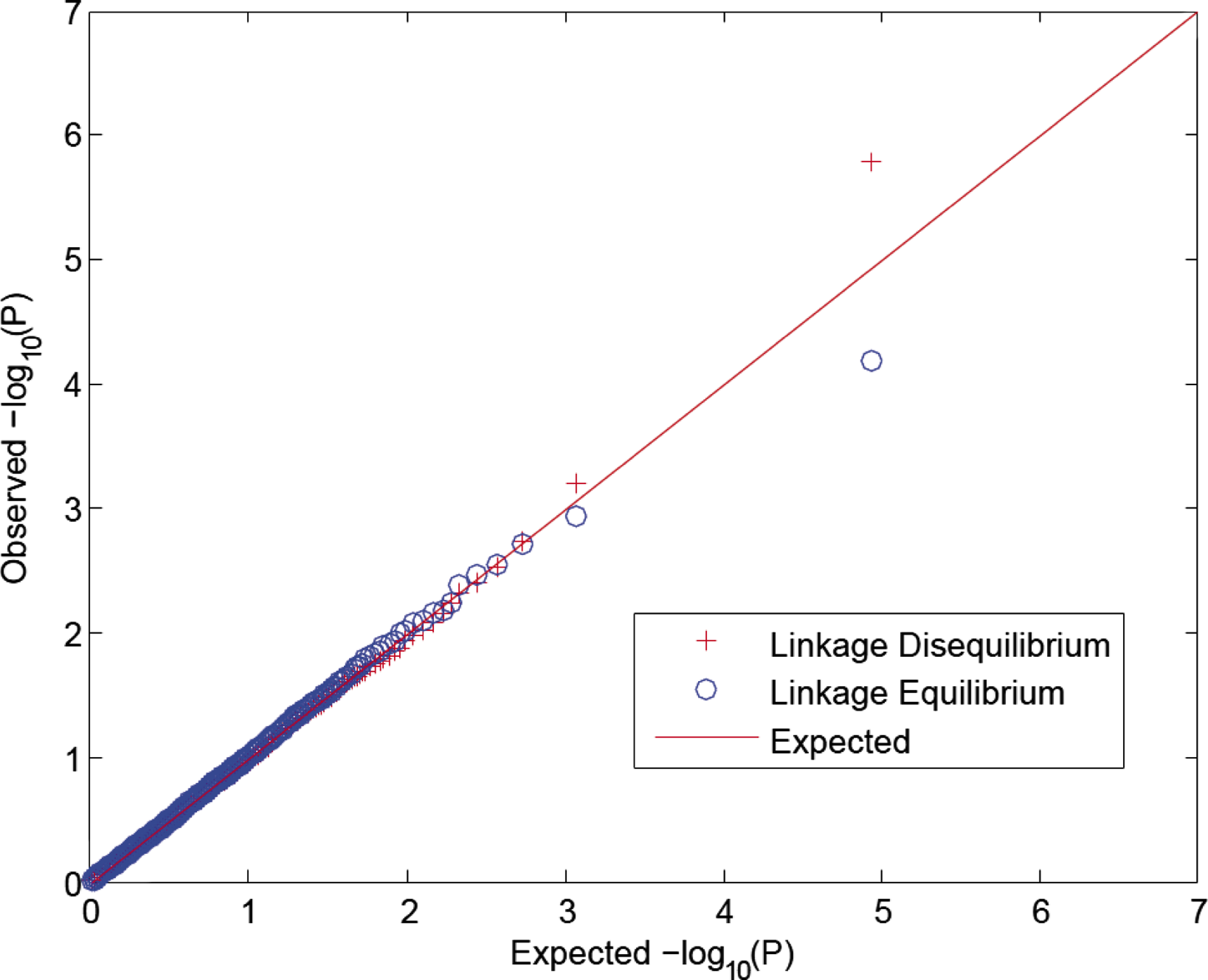

Supplement: Figure S3 — The effect of LD on SNP interaction analysis. 10,000 pairs of SNPs in LD (r2>0.5) and 10,000 pairs of SNPs not in LD (r2<0.01) were randomly selected over the genome and tested for interaction with the permutated color traits using F-test specified in the method section. The observed P values on the −log10(P) scale derived without LD (blue circle) or with LD (red plus) are plotted against the expected ones under the null distribution of no interaction. (0.39 MB TIF) [file pgen.1000934.s003.tif]

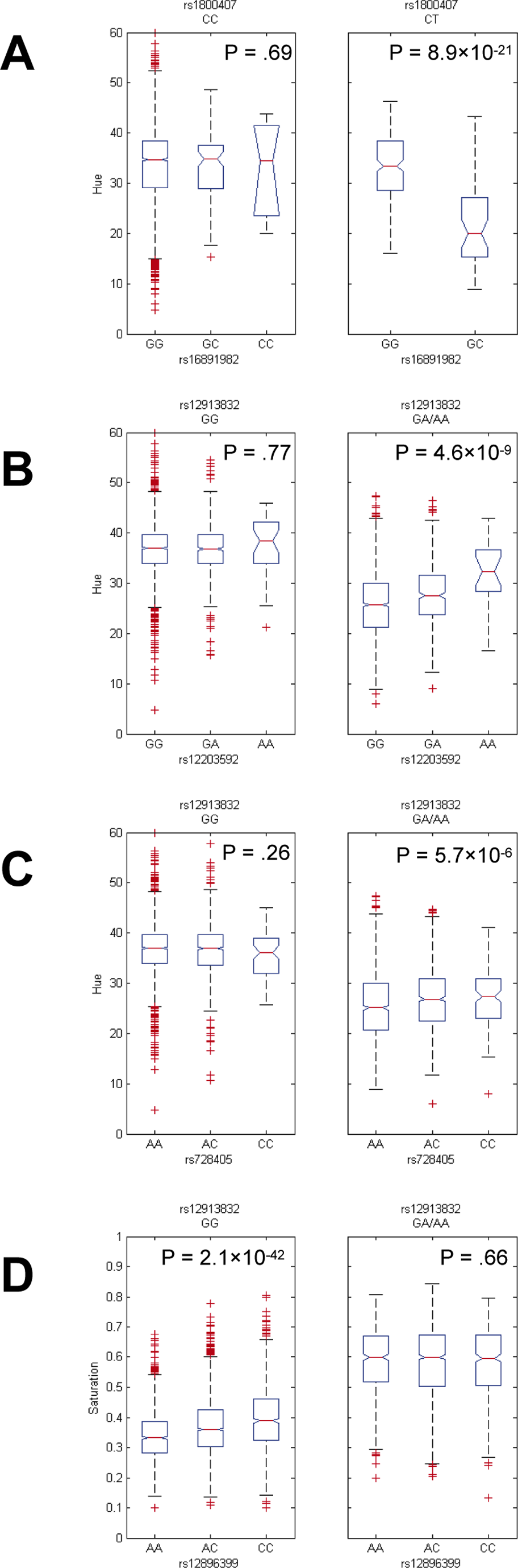

Supplement: Figure S4 — Significant SNP interactions on eye color. SNPs having significant interaction effect on eye color are depicted using box-and-whisker diagrams. Color H and S distributions are grouped by cross genotypes of 2 interacting SNPs. Distribution summaries include min-max range (black dotted vertical line), lower-upper 25% quartile range (blue box), and median (red line). Observations outside of 1.5 folds of the quartile range are indicated using red pluses. (0.34 MB TIF) [file pgen.1000934.s004.tif]
