# Supplementary material for: Digital Quantification of Human Eye Color Highlights Genetic Association of Three New Loci
Source: PLoS Genet. 2010 May 6;6(5):e1000934. doi: 10.1371/journal.pgen.1000934 (PMC2865509; doi:10.1371/journal.pgen.1000934)
Supplement: Table S1 — SNPs ascertained for pair-wise interaction analysis and P values from single SNP analysis in the Rotterdam Study (RS123). (0.11 MB DOC) [file pgen.1000934.s005.doc]

**Table S1. SNPs ascertained for pair-wise interaction analysis and P values from single SNP analysis**

|  |  |  |  |  |  |  |  |  |  |
| --- | --- | --- | --- | --- | --- | --- | --- | --- | --- |
|  |  |  |  | **P values** | | | | | |
| **Index** | **SNP** | **Chr** | **Gene** | **3color** | **5color** | **H** | **S** | **CHS1** | **CHS2** |
| 1 | rs3768056 | 1q42.3 | LYST | 0.073 | 0.015 | 0.368 | 8.3E-09 | 2.2E-04 | 3.4E-07 |
| 2 | rs9782955 | 1q42.3 | LYST | 0.168 | 0.027 | 0.374 | 6.0E-09 | 2.0E-04 | 2.3E-07 |
| 3 | rs16891982 | 5p15.33 | SLC45A2 | 2.9E-08 | 5.8E-07 | 9.3E-06 | 0.008 | 6.1E-05 | 0.035 |
| 4 | rs26722 | 5p15.33 | SLC45A2 | 2.9E-05 | 2.0E-06 | 4.4E-06 | 0.002 | 1.2E-05 | 0.080 |
| 5 | rs12203592 | 6p25.3 | IRF4 | 7.9E-05 | 8.7E-06 | 2.2E-05 | 1.6E-05 | 1.4E-06 | 0.896 |
| 6 | rs1540771 | 6p25.3 | IRF4 | 0.056 | 0.002 | 0.147 | 0.002 | 0.012 | 0.105 |
| 7 | rs791691 | 9p23 | TYRP1 | 1.7E-04 | 1.8E-06 | 4.0E-04 | 2.1E-08 | 2.9E-07 | 0.045 |
| 8 | rs702133 | 9p23 | TYRP1 | 4.7E-04 | 3.7E-06 | 0.001 | 4.6E-08 | 9.8E-07 | 0.030 |
| 9 | rs1408809 | 9p23 | TYRP1 | 5.4E-05 | 2.6E-07 | 4.2E-04 | 1.1E-08 | 2.2E-07 | 0.032 |
| 10 | rs10429629 | 9p23 | TYRP1 | 2.5E-07 | 5.2E-11 | 6.7E-07 | 1.2E-10 | 1.4E-10 | 0.187 |
| 11 | rs10809808 | 9p23 | TYRP1 | 1.3E-07 | 1.5E-10 | 3.3E-08 | 1.9E-11 | 5.8E-12 | 0.320 |
| 12 | rs1325127 | 9p23 | TYRP1 | 2.2E-09 | 1.1E-09 | 1.0E-07 | 1.3E-10 | 4.0E-11 | 0.363 |
| 13 | rs1408799 | 9p23 | TYRP1 | 9.6E-05 | 1.9E-06 | 4.9E-06 | 8.3E-08 | 2.3E-08 | 0.539 |
| 14 | rs10960751 | 9p23 | TYRP1 | 5.4E-09 | 1.3E-10 | 8.5E-09 | 1.2E-11 | 1.7E-12 | 0.425 |
| 15 | rs683 | 9p23 | TYRP1 | 1.2E-04 | 7.2E-06 | 1.4E-04 | 2.3E-06 | 1.6E-06 | 0.436 |
| 16 | rs1137134 | 9p23 | TYRP1 | 2.0E-07 | 2.8E-09 | 1.0E-06 | 4.2E-10 | 3.8E-10 | 0.231 |
| 17 | rs1042602 | 11q14.3 | TYR | 0.097 | 0.623 | 0.200 | 0.590 | 0.301 | 0.396 |
| 18 | rs11018528 | 11q14.3 | TYR | 1.7E-06 | 3.7E-10 | 1.2E-05 | 2.3E-10 | 1.8E-09 | 0.062 |
| 19 | rs10765198 | 11q14.3 | TYR | 2.0E-07 | 1.1E-11 | 1.0E-06 | 2.1E-13 | 6.8E-12 | 0.018 |
| 20 | rs1847134 | 11q14.3 | TYR | 2.2E-08 | 3.5E-13 | 1.1E-06 | 1.7E-12 | 2.1E-11 | 0.039 |
| 21 | rs1393350 | 11q14.3 | TYR | 4.3E-10 | 3.8E-14 | 3.1E-09 | 7.4E-18 | 3.3E-16 | 0.011 |
| 22 | rs1806319 | 11q14.3 | TYR | 1.5E-08 | 1.0E-09 | 4.5E-05 | 2.4E-08 | 5.8E-08 | 0.169 |
| 23 | rs1875565 | 11q14.3 | TYR | 0.005 | 5.8E-07 | 6.9E-04 | 6.2E-08 | 7.9E-07 | 0.049 |
| 24 | rs4904864 | 14q32.12 | SLC24A4 | 0.002 | 1.0E-09 | 0.028 | 2.9E-13 | 1.2E-07 | 1.3E-07 |
| 25 | rs4904866 | 14q32.12 | SLC24A4 | 2.3E-07 | 6.1E-20 | 5.9E-05 | 4.3E-23 | 8.2E-15 | 1.9E-09 |
| 26 | rs12896399 | 14q32.12 | SLC24A4 | 1.7E-07 | 4.8E-20 | 4.0E-05 | 2.0E-23 | 3.8E-15 | 2.1E-09 |
| 27 | rs4904868 | 14q32.12 | SLC24A4 | 7.6E-06 | 7.7E-16 | 1.4E-04 | 2.1E-18 | 2.2E-12 | 5.0E-07 |
| 28 | rs2594935 | 15q13.1 | OCA2 | 2.0E-27 | 1.4E-25 | 4.0E-21 | 1.9E-28 | 1.3E-27 | 0.040 |
| 29 | rs728405 | 15q13.1 | OCA2 | 1.1E-29 | 1.0E-26 | 5.2E-24 | 1.3E-35 | 4.1E-33 | 0.002 |
| 30 | rs1800407 | 15q13.1 | OCA2 | 5.6E-12 | 3.4E-11 | 3.1E-09 | 3.6E-10 | 9.0E-11 | 0.749 |
| 31 | rs3794604 | 15q13.1 | OCA2 | 3.0E-64 | 1.3E-46 | 3.8E-53 | 1.6E-42 | 7.7E-54 | 0.004 |
| 32 | rs4778232 | 15q13.1 | OCA2 | 1.3E-46 | 9.2E-36 | 3.7E-41 | 4.2E-37 | 4.4E-44 | 0.155 |
| 33 | rs1448485 | 15q13.1 | OCA2 | 8.7E-60 | 3.6E-42 | 3.4E-48 | 1.4E-38 | 8.3E-49 | 0.007 |
| 34 | rs8024968 | 15q13.1 | OCA2 | 1.2E-73 | 1.1E-53 | 1.4E-61 | 6.5E-50 | 7.7E-63 | 0.004 |
| 35 | rs1597196 | 15q13.1 | OCA2 | 1.5E-50 | 5.9E-37 | 4.2E-45 | 9.8E-41 | 2.2E-48 | 0.142 |
| 36 | rs7179994 | 15q13.1 | OCA2 | 8.9E-23 | 8.1E-21 | 2.4E-24 | 6.3E-26 | 3.3E-28 | 0.848 |
| 37 | rs4778138 | 15q13.1 | OCA2 | 4.6E-227 | 1.1E-199 | 1.1E-182 | 9.9E-164 | 3.0E-197 | 0.004 |
| 38 | rs4778241 | 15q13.1 | OCA2 | 4.4E-282 | 1.2E-257 | 1.9E-224 | 9.5E-227 | 8.1E-258 | 0.463 |
| 39 | rs7495174 | 15q13.1 | OCA2 | 1.1E-273 | 1.2E-231 | 1.0E-220 | 3.2E-202 | 2.0E-241 | 0.007 |
| 40 | rs1129038 | 15q13.1 | HERC2 | <1.0E-300 | <1.0E-300 | <1.0E-300 | <1.0E-300 | <1.0E-300 | 0.606 |
| 41 | rs12593929 | 15q13.1 | HERC2 | 6.6E-225 | 1.9E-192 | 1.3E-180 | 9.8E-173 | 2.5E-201 | 0.090 |
| 42 | rs12913832 | 15q13.1 | HERC2 | <1.0E-300 | <1.0E-300 | <1.0E-300 | <1.0E-300 | <1.0E-300 | 0.602 |
| 43 | rs7183877 | 15q13.1 | HERC2 | 2.0E-142 | 8.3E-134 | 2.6E-103 | 1.1E-128 | 4.9E-131 | 0.005 |
| 44 | rs11635884 | 15q13.1 | HERC2 | 9.3E-30 | 1.9E-31 | 3.8E-36 | 1.3E-17 | 2.6E-29 | 7.7E-10 |
| 45 | rs3935591 | 15q13.1 | HERC2 | <1.0E-300 | <1.0E-300 | <1.0E-300 | <1.0E-300 | <1.0E-300 | 0.893 |
| 46 | rs7170852 | 15q13.1 | HERC2 | <1.0E-300 | <1.0E-300 | <1.0E-300 | <1.0E-300 | <1.0E-300 | 0.778 |
| 47 | rs8041209 | 15q13.1 | HERC2 | 2.6E-211 | 1.2E-178 | 4.6E-171 | 9.6E-167 | 2.3E-192 | 0.192 |
| 48 | rs8028689 | 15q13.1 | HERC2 | 2.2E-210 | 1.3E-178 | 1.1E-172 | 2.7E-168 | 3.3E-194 | 0.188 |
| 49 | rs2240203 | 15q13.1 | HERC2 | 1.1E-209 | 1.0E-176 | 1.4E-171 | 2.6E-167 | 5.4E-193 | 0.194 |
| 50 | rs2240202 | 15q13.1 | HERC2 | 6.5E-208 | 2.1E-177 | 9.8E-171 | 4.0E-167 | 2.1E-192 | 0.220 |
| 51 | rs916977 | 15q13.1 | HERC2 | <1.0E-300 | <1.0E-300 | <1.0E-300 | <1.0E-300 | <1.0E-300 | 0.927 |
| 52 | rs16950979 | 15q13.1 | HERC2 | 9.6E-209 | 5.2E-178 | 2.1E-171 | 5.0E-168 | 2.6E-193 | 0.229 |
| 53 | rs2346050 | 15q13.1 | HERC2 | 4.6E-212 | 6.6E-180 | 4.3E-173 | 4.8E-169 | 7.2E-195 | 0.201 |
| 54 | rs16950987 | 15q13.1 | HERC2 | 7.5E-213 | 1.4E-180 | 4.4E-174 | 4.0E-170 | 4.7E-196 | 0.205 |
| 55 | rs1667394 | 15q13.1 | HERC2 | <1.0E-300 | <1.0E-300 | <1.0E-300 | <1.0E-300 | <1.0E-300 | 0.932 |
| 56 | rs12592730 | 15q13.1 | HERC2 | 6.5E-208 | 2.1E-177 | 9.8E-171 | 4.0E-167 | 2.1E-192 | 0.220 |
| 57 | rs1635168 | 15q13.1 | HERC2 | 7.5E-219 | 2.6E-187 | 1.3E-181 | 3.8E-172 | 1.5E-201 | 0.062 |
| 58 | rs7219915 | 17q25.3 | NPLOC4 | 6.9E-04 | 2.2E-09 | 4.6E-09 | 6.4E-09 | 4.8E-11 | 0.730 |
| 59 | rs9894429 | 17q25.3 | NPLOC4 | 0.002 | 3.4E-07 | 6.3E-09 | 6.1E-08 | 2.4E-10 | 0.489 |
| 60 | rs6058017 | 21q22.13 | TTC3 | 0.314 | 0.890 | 0.061 | 0.353 | 0.109 | 0.221 |
| 61 | rs2252893 | 21q22.13 | TTC3 | 0.003 | 1.6E-04 | 3.6E-07 | 6.9E-09 | 9.1E-10 | 0.618 |
| 62 | rs2835621 | 21q22.13 | TTC3 | 0.003 | 1.7E-04 | 2.2E-07 | 8.7E-09 | 7.2E-10 | 0.723 |
| 63 | rs2835630 | 21q22.13 | TTC3 | 0.003 | 8.1E-05 | 1.4E-06 | 1.8E-08 | 4.0E-09 | 0.548 |
| 64 | rs7277820 | 21q22.13 | DSCR9 | 0.002 | 6.6E-05 | 5.9E-07 | 1.6E-08 | 2.1E-09 | 0.654 |
|  |  |  |  |  |  |  |  |  |  |
| P values are adjusted for the effect of HERC2 rs12913832 except the chromosome 15q13.1 region | | | | | | | | | |
