# Supplementary material for: Digital Quantification of Human Eye Color Highlights Genetic Association of Three New Loci
Source: PLoS Genet. 2010 May 6;6(5):e1000934. doi: 10.1371/journal.pgen.1000934 (PMC2865509; doi:10.1371/journal.pgen.1000934)
Supplement: Text S1 — Interaction analysis. (0.04 MB DOC) [file pgen.1000934.s006.doc]

**Supporting online material**

**Digital quantification of human eye color highlights genetic association of three new loci**

Fan Liu^1^, Andreas Wollstein^1,2^, Pirro G. Hysi^3^, Georgina A. Ankra-Badu^3^, Timothy D. Spector^3^, Daniel Park^4^, Gu Zhu^4^, Mats Larsson^4^, David L. Duffy^4^, Grant W. Montgomery^4^, David A. Mackey^5^, Susan Walsh^1^, Oscar Lao^1^, Albert Hofman^6^, Fernando Rivadeneira^6,7^, Johannes R. Vingerling^6,8^, André G. Uitterlinden^6,7^, Nicholas G. Martin^4^, Christopher J. Hammond^3^, and Manfred Kayser^1^

^1^Department of Forensic Molecular Biology, ^6^Department of Epidemiology, ^7^Department of Internal Medicine, ^8^Department of Ophthalmology, all from the Erasmus University Medical Center Rotterdam, The Netherlands

^2^Cologne Center for Genomics (CCG), University of Cologne, Cologne, Germany

^3^Department of Twin Research & Genetic Epidemiology, King’s College London, London, United Kingdom

^4^Queensland Institute of Medical Research, Brisbane, Australia

^5^ Centre for Ophthalmology and Visual Science, Lions Eye Institute, University of Western Australia, Perth, Australia

Correspondence to:

Prof. Dr. Manfred Kayser, Department of Forensic Molecular Biology, Erasmus University Medical Center Rotterdam, P. O. Box 2040, 3000 CA Rotterdam, The Netherlands. E-mail: m.kayser@erasmusmc.nl, Tel: +31-10-7038073, Fax: +31-10-7044575.

**Interaction analysis**

We tested pair-wise interactions between 64 SNPs from 7 previously known genes (*HERC2*, *OCA2*, *SLC2A4*, *TYR*, *TYRP1*, *SLC45A2*, *IRF4*) and the 3 newly identified loci (1q42.3, 17q25.3, 21q22.13) and for H and S (Table S1). The interactions were tested at the multiplicative scale between each pair of SNPs by comparing two models with and without the interaction term using F-test. Let a column vector *y* denote the color trait residuals after regressing out the effects of known factors. Let a *n*-by-3 matrix *X_0_* denote individual genotypes, where the 1^st^ column is constant of ones, 2^nd^ and 3^rd^ columns are the number of minor alleles minus 1 of the 2 SNPs under testing (-1, 0, 1). Let *X_1_* contain an additional column of the interaction term at the multiplicative scale.

The total sum of squares is

$SSt=y^{T}y-n\bar{y}^{2}$.

The residual sum of squares in the model without the interaction term is

$SSR0=((X_{0}^{T}X_{0})^{-1}X_{0}^{T}y)^{T}X_{0}^{T}y-n\bar{y}^{2}$;

and the residual sum of squares in the model with the interaction term is

$SSR1=((X_{1}^{T}X_{1})^{-1}X_{1}^{T}y)^{T}X_{1}^{T}y-n\bar{y}^{2}$.

The *F* value can be derived based on the sum of squares,

$F=\frac{SSR1-SSR0}{(SSt-SSR1)/(n-1)}$,
which follows the *F* distribution with 1 and *n*-1 degrees of freedom under the null hypothesis of no interaction. A next round analysis is performed by adjusting for the effect of significant interactions in a previous round until no more significant interaction was detected. The significance threshold was defined at the level of P = 10^-5^.

Because some of the SNPs tested are in high LD, we investigated the effect of LD on significant findings. We randomly selected over autonomies 10,000 pairs of SNPs in LD (*r^2^* > 0.5) and 10,000 pairs of SNPs not in LD (*r^2^* < 0.01) and tested for interaction with permutated color traits using the specified F-test. The observed test statistics, regardless to the presence of LD, did not deviate from the expected ones under the null distribution of no interaction (Figure S3). Thus, it is unlikely that the significant interactions identified in the current study are spurious due to LD between the SNPs.

We detected significant pair-wise interactions between SNPs in the *HERC2*, *OCA2*, *SLC24A4*, and *IRF4* genes (Figure S2). The most significant interactions were between *OCA2* rs1800407 and *SLC45A2* rs16891982 (P = 2.7×10^-6^ for H, P = 7.3×10^-5^ for S), *HERC2* rs12913832 and *IRF4* rs12203592 (P = 6.1×10^-9^ for H, P = 1.4×10^-6^ for S), *HERC2* rs12913832 and *OCA2* rs728405 (P = 2.1×10^-6^ for H only), and *HERC2* rs12913832 and *SLC24A4* rs12896399 (P = 1.9×10^-14^ for S only). After these effects were adjusted, no additional SNP interactions were significant. Furthermore, we examined the distributions of H and S stratified by genotypes of the interacting SNPs. The effect of rs16891982 on H was only seen in rs1800407 CT carriers (P = 8.9×10^-21^) but not in CC carriers (P = .69) (Figure. S4A). The effects of rs12203592 and rs728405 on H were seen in rs12913832 GA/AA carriers (P < 5.7×10^-6^) but not in GG carriers (P > .26) (Figures S4B and S4C). The *SLC24A4* rs12896399 showed a highly significant effect on S in *HERC2* rs12913832 GG carriers (P = 2.1×10^-42^) but not in GA/AA carriers (P = .66) (Figure. S4D).
